# Relationship between TRAF6 and deterioration of HCC: an immunohistochemical and in vitro study

**DOI:** 10.1186/s12935-016-0352-z

**Published:** 2016-09-29

**Authors:** Jian-jun Li, Jie Luo, Jing-ning Lu, Xiao-na Liang, Yi-huan Luo, Yong-ru Liu, Jie Yang, Hua Ding, Gui-hui Qin, Li-hua Yang, Yi-wu Dang, Hong Yang, Gang Chen

**Affiliations:** 1 Department of General Surgery, Western Branch, First Affiliated Hospital of Guangxi Medical University, Nanning, 530021 Guangxi Zhuang Autonomous Region People’s Republic of China; 2 Department of Medical Oncology, First Affiliated Hospital of Guangxi Medical University, 6 Shuangyong Road, Nanning, 530021 Guangxi Zhuang Autonomous Region People’s Republic of China; 3 Department of Hepatobiliary Surgery, First Affiliated Hospital of Guangxi Medical University, 6 Shuangyong Road, Nanning, 530021 Guangxi Zhuang Autonomous Region People’s Republic of China; 4 Department of Pathology, First Affiliated Hospital of Guangxi Medical University, 6 Shuangyong Road, Nanning, 530021 Guangxi Zhuang Autonomous Region People’s Republic of China; 5 Department of Pharmacology, School of Pharmacy, Guangxi Medical University, Nanning, 530021 Guangxi Zhuang Autonomous Region People’s Republic of China; 6 Department of Radiotherapy, First Affiliated Hospital of Guangxi Medical University, 6 Shuangyong Road, Nanning, 530021 Guangxi Zhuang Autonomous Region People’s Republic of China; 7 Department of Ultrasonography, First Affiliated Hospital of Guangxi Medical University, 6 Shuangyong Road, Nanning, 530021 Guangxi Zhuang Autonomous Region People’s Republic of China

**Keywords:** TRAF6, Hepatocellular carcinoma, Clinicopathological parameters, Proliferation, Apoptosis, Caspase

## Abstract

**Objective:**

To explore the relationship between tumor necrosis factor receptor-associated factor 6 (TRAF6) and the clinicopathological features in HCC as well as its biological function.

**Methods:**

Totally, 412 liver tissues were collected, including 171 hepatocellular carcinoma (HCC) and their corresponding non-tumor tissues, 37 cirrhosis and 33 normal liver tissues. The expression of TRAF6 was assessed by immunohistochemistry. Then, analysis of the correlations between TRAF6 expression and clinicopathological parameters in HCC was conducted. Furtherer, in vitro experiments on HepG2 and Hep3B cells were performed to validate the biological function of TRAF6 on HCC cells. TRAF6 siRNA was transfected into HepG2 and Hep3B cell lines and TRAF6 expression was evaluated with RT-qPCR and western blot. The assays of cell viability, proliferation, apoptosis and caspase-3/7 activity were carried out to investigate the effects of TRAF6 on HCC cells with RNA interference. Cell viability was assessed with Cell Titer-Blue kit. Cell proliferation was tested with MTS kit. Cell apoptosis was checked through morphologic detection with fluorescence microscope, as well as caspase-3/7 activity was measured with fluorogenic substrate detection.

**Results:**

The positive expression rate of TRAF6 protein was 49.7 % in HCC, significantly higher than that of normal liver (12.1 %), cirrhosis (21.6 %) and adjacent non-cancerous tissues (36.3 %, all *P* < 0.05). Upregulated TRAF6 was detected in groups with metastasis (Z = −2.058, *P* = 0.04) and with low micro-vessel density (MVD) expression (Z = −2.813, *P* = 0.005). Spearman correlation analysis further showed that the expression of TRAF6 was positively correlated with distant metastasis (r = 0.158, *P* = 0.039) and negatively associated with MVD (r = −0.249, *P* = 0.004). Besides, knock-down of TRAF6 mRNA in HCC cell lines HepG2 and Hep3B both resulted in cell viability and proliferation inhibition, also cell apoptosis induction and caspase-3/7 activity activation.

**Conclusions:**

TRAF6 may contribute to metastasis and deterioration of the HCC via influencing cell growth and apoptosis. Thus, TRAF6 might become a predictive and therapeutic biomarker for HCC.

## Background

Hepatocellular carcinoma (HCC) is the fifth most frequent malignancy in the world [1]. With a mortality rate of about one million per year, it ranks the third leading cause of cancer deaths worldwide [2]. Roughly, 80 % HCC patients, among all cases, live in the Asia–Pacific region and Sub-Saharan Africa area while the incidence has also increased markedly in Europe and the United States [3–5]. It has been estimated that there would be 35,660 new cases with liver cancer in America in 2015 [6]. Several environmental factors are considered to be contributed to the main causes of HCC, such as long-time exposure to aflatoxin B1, hepatitis B and C viral infections and alcohol abuse [7–9]. Despite great progress has been made in diagnosis and therapy for HCC, there are limitations in early detection, and the patients in advanced stage, when diagnosed, generally face a dismal prognosis and a high incidence of recurrence [10, 11]. At present, α-fetoprotein (AFP) is the common clinical diagnostic criteria for HCC [12], especially in Asia. However, its specificity and sensitivity are not satisfied [13]. Therefore, identifying reliable novel biomarkers for the early screening and prediction of HCC arouses our interest.

Tumor necrosis factor receptor-associated factors (TRAFs) are a group of cytoplasmic adapter proteins which link a wide range of cell surface receptors. TRAFs mediate intracellular signal transduction, including tumor necrosis factor (TNF), toll-like receptor (TLR), interleukin-1 receptor (IL-1R) and superfamily transforming growth factor-β (TGF-β) [14, 15]. As a result, TRAFs lead to the activation of mitogen-activated protein and nuclear factor kappa B (NF-κB) kinases [16]. Additionally, TRAFs also interact with varieties of proteins which regulate receptor-induced cell death and survival [16]. Therefore, TRAF-mediated signal cascades may directly cause the receptor-induced apoptosis and interfere with cell survival.

Among the six TRAF family members, TRAF6 is unique. TRAF6 is involved in a variety of physiological processes, including congenital immunity, adaptive immunity, and inflammation [17–19]. TRAF6, functioning as a signal transducer, is a key activator of NF-κB pathway which activates inhibitor of IκB kinase in response to proinflammatory cytokines [20, 21]. In recent years TRAF6 has been found to play some roles in carcinogenesis in some cancers, such as lung cancer [22], glioblastoma [23], and squamous cell carcinoma [24]. However, none of the studies have cast light upon the expression and function of TRAF6 and its association with HCC. Thus, in order to investigate the relationship between TRAF6 and clinicopathological parameters of HCC, whether TRAF6 plays a role in the proliferation and apoptosis process of HCC or not, we carried out the current study, quantifying the TRAF6 differential expression in liver tissues with continuous pathological states, which could lead to potential development of HCC using immunohistochemistry (IHC) technique, and testing the effects of silencing TRAF6 in HCC HepG2 and Hep3B cell lines with in vitro experiments.

## Methods

### Study design and experimental grouping design

The study was concentrated on the following three parts. Firstly, the expression of TRAF6 was compared between group of HCC and non-HCC liver tissues. Groups of non-HCC liver tissues included group of para-tumor liver tissue, group of cirrhosis liver tissue and group of normal liver tissue. Secondly, the relationship between TRAF6 and clinicopathological data of HCC patients was analyzed. Subgroup classification was based on different status of each clinicopathological data, including age, gender, tumor differentiation, tumor size, number of tumor nodes, metastasis, clinical TNM stages, status of portal vein tumor embolus, vaso-invasion condition, capsular infiltration, hepatitis C virus (HCV) and hepatitis B virus (HBV) infection status, AFP expression, cirrhosis, as well as nm23, p53, p21, vascular endothelial growth factor (VEGF), Ki-67 expression and micro-vessel density (MVD) level as stained by CD34. Thirdly, in vitro experiments on HCC cell lines, including HepG2 and Hep3B, were performed to validate the biological function of TRAF6 on HCC cells. The cell viability, cell proliferation, cell apoptosis and Caspase-3/7 activity were compared between the group of negative controls and the group transfected with TRAF6 siRNA.

### Tissue samples

HCC tissues (n = 171) and their corresponding para-cancer tissues, 37 cirrhosis liver tissues and 33 normal liver tissues were collected from patients or autopsies admitted to the First Affiliated Hospital, Guangxi Medical University from March 2010 to December 2012. All these HCC tissues were taken from patients with primary HCC, who had not received any other treatment before surgery. The clinicopathological data of 171 HCC patients were listed above. All the formalin-fixed, paraffin embedded (FFPE) liver tissues were evaluated retrospectively, and their diagnoses were confirmed by two experienced pathologists independently. Besides, enzyme-linked immunosorbent assay (ELISA) was used to measure AFP level in sera and IHC was applied to detect nm23, p53, p21, VEGF, Ki-67 and MVD. All the features above were recorded. The study was approved by the Ethical Committee of the First Affiliated Hospital of Guangxi Medical University. Informed consent was signed by all of the patients who participated in the study. Related research procedure complied with the Helsinki Declaration.

### Immunohistochemistry

Immunohistochemical technique was used to detect the expression of TRAF6. The TRAF6 antibody obtained from Santa Cruz Biotechnology, lnc. (D-10, sc-8409, 1:300 dilution, Heidelberg, Germany) was applied to antigen–antibody interaction. TRAF6 Antibody (D-10) is a mouse monoclonal IgG1 provided at 200 µg/ml and it is raised against amino acids 1–274 mapping at the N-terminus of TRAF6 of human origin. All the experimental operations were completed strictly according following instructions. Two pathologists reviewed the H&E sections and scored the staining independently, without knowing the status of the samples. The scoring standards were defined as follows [25]: (1) the staining intensity was marked from 0 to 3 points, 0 for no staining, 1 for weak, 2 for moderate and 3 for strong. (2) The positive expression rate ranged from 0 to 4 points, 0 stands for no staining, 1 for <25 %; 2 for 26 to 50 %, 3 for 51 to 75 %, and 4 for >75 %. (3) The points of staining intensity plus the points of positive expression rate resulted in the final expression score.

### Culture and transfection of the cell lines

Human HCC cell lines HepG2 and Hep3B were purchased from Shanghai Institute of Cell Biology of Chinese Academy of Sciences. Cells were cultured in the RPMI1640 medium with 10 % fetal bovine serum at 37 ℃ in a 5 % CO_2_ incubator. HepG2 and Hep3B cells were inoculated in 96 or 24-well plates and cultured for 24 h. Cell confluence was controlled at 50 to 60 % when cells were transfected with TRAF6 siRNA with Lipofectamine 2000 reagent (Invitrogen). After the transfection, we chose 0, 5 and 10 days as time points to detect the effects of TRAF6 siRNA on the malignant phenotypes of HCC cells.

### Real time RT-qPCR and Western blot

TRAF6 expression of the HepG2 and Hep3B cell lines transfected with TRAF6 siRNA was measured with real time RT-qPCR and western blot.

The TRAF6 mRNA level was determined after 5 and 10 days post-transfection by RT-qPCR. Briefly, total cellular RNA isolation was performed with RNeasy Mini kit (Qiagen, Hilden, Germany). RNA concentrations were evaluated with a ND-2000 NanoDrop (Thermo Fisher Scientific, Wilmington, Delaware USA). Two hundred nanogram of cellular RNA was converted to cDNA with a High Capacity cDNA Reverse Transcription Kit (Applied Biosystems) in a total volume of 10 μl. The primers for TRAF6 were: forward 5′- AGGGACCCAGCTTTCTTTGT-3′ and reverse 5′- GCCAAGTGATTCCTCTGCAT-3′. The primers for GAPDH were: forward 5′-TGAAGGTCGGAGTCAACGGATTTGGT-3′ and reverse 5′-CATGTGGGCCAT GAGGTCCACCAC-3′. RT-qPCR analysis was carried out on a LightCycler 1.5 using the Faststart DNA master SYBR green mastermix. Quantitative values were obtained from the PCR quantification cycle number (Cq) at which point the increase in signal for the PCR product was exponential. The target mRNA abundance in each sample was normalized to its GAPDH mRNA level as ΔCq = Cq_TRAF6_−Cq_GAPDH_. The value ΔΔCq was defined as the difference with a mock transfected control. Experiments were performed in triplicate. The knockdown ratio of TRAF6 mRNA expression was calculated with the formula: (1−1/2^ΔΔCq^) × 100 %.

For western blot, totally, 25 μg proteins obtained from the cell lines were added in 8 % SDS-PAGE separation gel. Then the mixture was transferred to nitrocellulose membranes and probed with primary antibodies against TRAF6 (D-10, sc-8409, dilution: 1:500, Santa Cruz Biotechnology). After incubating with horseradish peroxidase (HRP)-conjugated secondary antibodies (D-3004), enhanced chemiluminescence detection was performed to identify the expression levels of TRAF6 protein.

### Cell viability assay

The assay was conducted with Cell Titer-Blue kit (G8080, Promega, USA). The detection time points were set on 0th, 5th and 10th day after transfection. The cell viability was determined with FL600 fluorescent detector (Bio-Tek, USA) at 560 nm or 590 nm.

### Cell proliferation assay

Cell proliferation was measured with MTS kit according to manufacturer’s instructions on 0th, 5th and 10th day post transfection. The medium was cleared out and washed with phosphate-buffered saline twice. Fetal bovine serum of 10 % and MTS reagents were added to subset of wells. After the cells were incubated for 2 h, absorbance at 490 nm was measured with a microplate reader (Thermo).

### Apoptosis assay

Cells were washed with cold phosphate-buffered saline and stained with 1 mg/ml Hoechst 33342 and 1 mg/ml PI for 15 min, fluorescence microscope (ZEISS Axiovert 25) was then used to observe the cellular morphology and staining changes. Ten different photos were taken under 200 times visual sight. The judging criterion was as follows: (1) Positive Hoechst 33342 staining and negative PI staining was found in living cells with complete shape. (2) Early apoptotic cells were stained with positive Hoechst and negative PI, containing blue apoptotic bodies, while apoptotic cells in the late phase were stained with negative Hoechst and positive PI, containing red apoptotic bodies. (3) Necrotic cells were those cells with negative PI staining but with none apoptotic bodies.

### Caspase-3/7 activity assay

After the cell viability assay was completed, we carried out the experiments on the same 96-well were to detect caspase-3/7 activity by adding caspase-3/7 reagents. Then the cells were incubated for an hour. Fluorescence intensity was measured at 499 nm or 512 nm with FL600 fluorescent detector (Bio-Tek, USA).

### Statistical analysis

SPSS21.0 was employed for statistical analysis. Kruskal–Wallis H tests were applied to analyze the status of TRAF6 protein expression among normal, cirrhosis, para-cancer and HCC tissues as well as their pathological grades. Mann–Whitney U tests were performed to identify the expression of TRAF6 in different subgroups with other clinicopathological features, including age, gender, tumor size, tumor nodes, metastasis, clinical TNM staging and so on. Correlation between the TRAF6 expression and clinicopathological features was detected with Spearman analysis. Furthermore, we performed receiver operating characteristic (ROC) curve to analyze the diagnostic ability of TRAF6 expression. Comparisons among groups of blank control, siRNA control and TRAF6 siRNA were performed with One-Way ANOVA or Kruskal–Wallis H tests. A *P* value less than 0.05 was considered statistically significant.

## Results

### TRAF6 expression in HCC tissues and non-cancer tissues

**Fig. 1:**
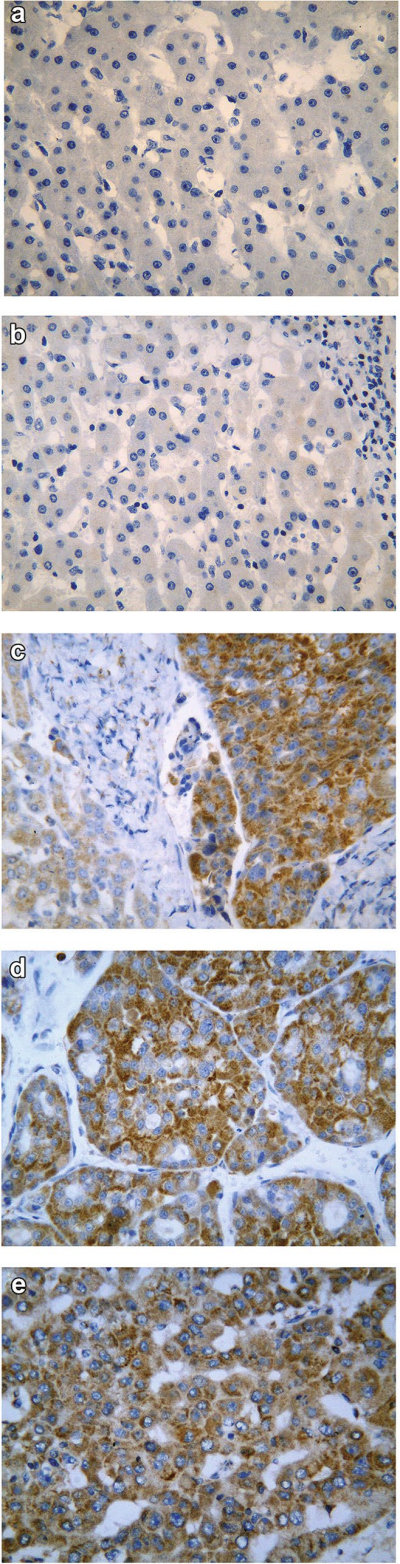
Expression pattern of TRAF6 in different liver tissues. Immunohistochemistry was performed to detect the TRAF6 expression in normal liver tissues (**a**), cirrhosis (**b**), para-tumor liver tissues (**c**, *left side*), and hepatocellular carcinoma tissues (**c**, *right side*, **d** and **e**). ×400

The positive expression rate of TRAF6 protein was 49.7 % (85/171) in HCC tissues, significantly higher than para-carcinoma liver tissues (36.3 %, *P* = 0.012), cirrhosis tissues (21.6 %, *P* = 0.002), and normal liver tissues (12.1 %, *P* < 0.001, Table 1, Fig. 1). Difference was also found between para-carcinoma liver tissues and normal liver tissues (Z = −2.707, *P* = 0.007). However, no statistical significance was observed when comparing cirrhotic liver tissues with para-carcinoma tissues or with normal tissues (*P* > 0.05).

**Table 1:** Expression of TRAF6 Protein in different tissues

Tissue	Patients(N)	TRAF6 negative N (%)	TRAF6 positive N (%)	*Z*	*P*
*HCC VS*					
HCC tissue	171	86 (50.3)	85 (49.7)	_	_
Para-carcinoma tissue	171	109 (63.7)	62 (36.3)	−2.509	0.012
Cirrhotic tissue	37	29 (78.4)	8 (21.6)	−3.108	0.002
Normal liver tissue	33	29 (87.9)	4 (12.1)	−3.976	0.001
*Para VS*					
Cirrhotic tissue	37	29 (78.4)	8 (21.6)	−1.704	0.088
Normal liver tissue	33	29 (87.9)	4 (12.1)	−2.707	0.007
*Cirrhosis VS*					
Normal liver tissue	33	29 (87.9)	4 (12.1)	−1.045	0.296

### Relationships between TRAF6 expression and clinicopathological parameters in HCC

**Fig. 2:**
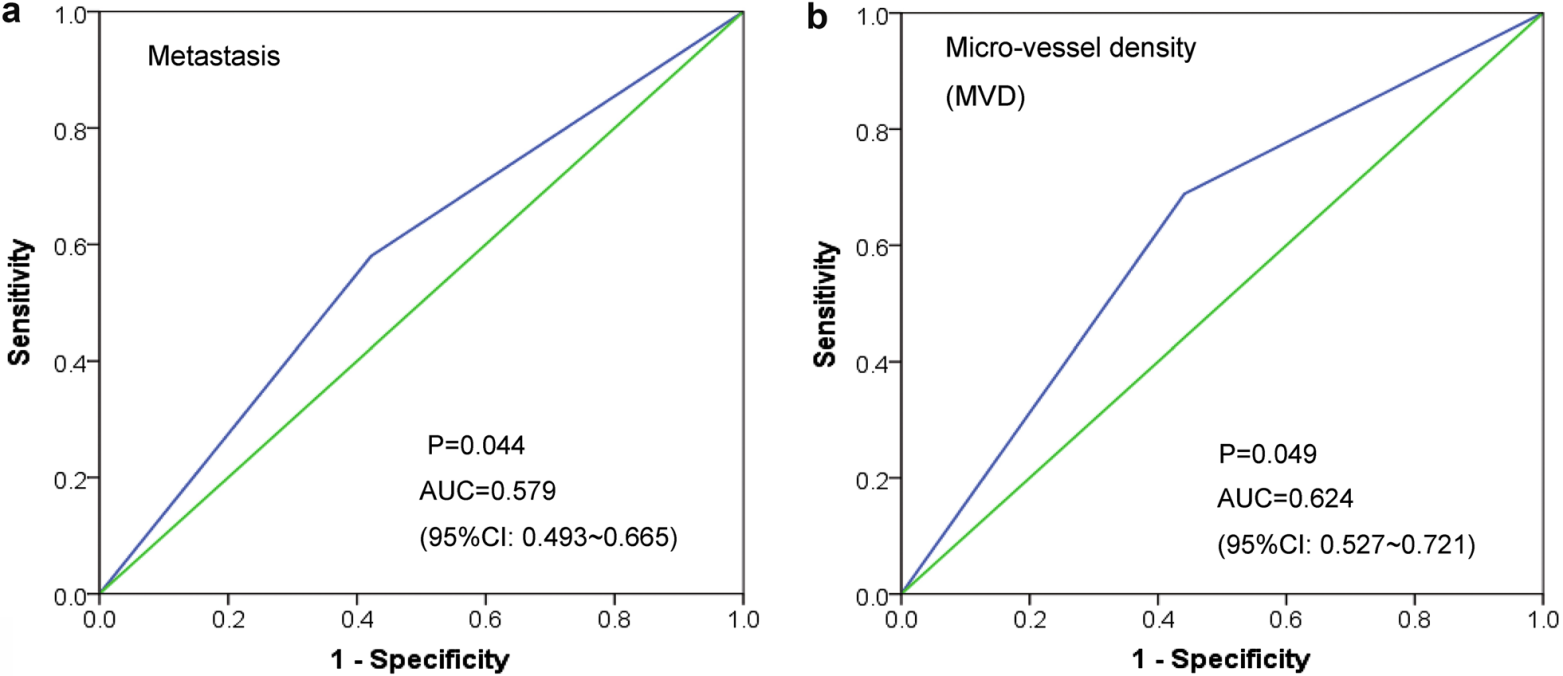
Receiver operating characteristic (ROC) curve of TRAF6 expression in hepatocellular carcinoma (HCC) tissues. The area under ROC curve (AUC) of TRAF6 was calculated to to predict the metastatic status and micro-vessel density (MVD) in HCC tissues

Correlations between the expression of TRAF6 protein and clinicopathological features were analyzed. The TRAF6 level was higher in patients with metastasis than those without. Increasing positive expression rate of TRAF6 was discovered in the cases with low level of MVD (Table 2). The area under ROC curve (AUC) of TRAF6 expression to predict metastasis and MVD were 0.579 (95 % CI 0.493–0.665) and 0.624 (95 % CI 0.527–0.721, Fig. 2), respectively. Moreover, Spearman correlation results showed that the expression of TRAF6 was significantly associated with distant metastasis (r = 0.158, *P* = 0.039) and MVD (r = −0.249, *P* = 0.004). However, no significant correlation was found between the expression of TRAF6 and other parameters.

**Table 2:** Relationship between the expression of TRAF6 and clinicopathological features in HCC

Clinicopathological parameters		*n*	TRAF6 negative n (%)	TRAF6 positive n (%)	*Z*	*P*	*Correlation*	
							*r*	*p*
*Age*								
<50		67	37 (55.2)	30 (44.8)	−1.032	0.302		
≥50		104	49 (47.1)	55 (52.9)				
*Gender*								
Male		153	76 (49.7)	77 (50.3)	−0.471	0.638	−0.036	0.639
Female		18	10 (55.6)	8 (44.4)				
*Differentiation*								
High		20	14 (70.0)	6 (30.0)	3.685*	0.158	0.116	0.130
Moderate		98	48 (49.0)	50 (51.0)				
Low		53	24 (45.3)	29 (54.7)				
*Size*								
<5 cm		58	34 (58.6)	24 (41.4)	−1.556	0.120	0.119	0.120
≥5 cm		113	52 (46.0)	61 (54.0)				
*Tumor nodes*								
Single		68	29 (42.6)	39 (57.4)	−0.370	0.711	−0.033	0.713
Multiple		61	28 (45.9)	33 (54.1)				
*Metastasis*								
–		90	52 (57.8)	38 (42.2)	−2.058	0.040	0.158	0.039
+		81	34 (42.0)	47 (58.0)				
*Clinical TNM stage*								
I ~ II		48	29 (60.4)	19 (39.6)	−1.649	0.099	0.126	0.099
III ~ IV		123	57 (46.3)	66 (53.7)				
*Portal vein tumor embolus*								
–		84	37 (44.0)	47 (56.0)	−0.043	0.966	−0.004	0.966
+		45	20 (44.4)	25 (55.6)				
*Vaso-invasion*								
–		77	34 (44.2)	43 (55.8)	−0.008	0.993	0.000	0.993
+		52	23 (44.2)	29 (55.8)				
*Tumor capsular infiltration*								
Yes		61	27 (44.3)	34 (55.7)	−0.016	0.987	0.001	0.987
No		68	30 (44.1)	38 (55.9)				
*HCV*								
–		87	40 (46.0)	47 (54.0)	−0.587	0.557	0.052	0.559
+		42	17 (40.5)	25 (59.5)				
*HBV*								
–		24	9 (37.5)	15 (62.5)	−0.728	0.466	−0.064	0.469
+		105	48 (45.7)	57 (54.3)				
*AFP*								
–		56	24 (42.9)	32 (57.1)	−0.748	0.455	0.072	0.457
+		54	27 (50.0)	27 (50.0)				
*Cirrhosis*								
–		74	33 (44.6)	41 (55.4)	−1.298	0.194	0.100	0.195
+		97	53 (54.6)	44 (45.4)				
*nm23*								
–		25	7 (28.0)	18 (72.0)	−1.808	0.071	−0.160	0.070
+		104	50 (48.1)	54 (51.9)				
*P53*								
–		54	25 (46.3)	29 (53.7)	−0.408	0.683	0.036	0.685
+		75	32 (42.7)	43 (57.3)				
*P21*								
–		84	36 (42.9)	48 (57.1)	−0.414	0.679	−0.037	0.681
+		45	21 (46.7)	24 (53.3)				
*VEGF*								
–		33	13 (39.4)	20 (60.6)	−0.640	0.522	−0.057	0.524
	+	96	44 (45.8)	52 (54.2)				
*Ki-67 LI*								
Low		62	26 (41.9)	36 (58.1)	−0.803	0.422	−0.073	0.424
High		61	30 (49.2)	31 (50.8)				
*MVD*								
Low		61	19 (31.1)	42 (68.9)	−2.813	0.005	−0.249	0.004
High		68	38 (55.9)	30 (44.1)				

* Kruskal–Wallis H test; − Negative; + Positive; *LI* label index; *MVD* micro-vessel density

### Inhibitory effect of TRAF6 on cell growth

**Fig. 3:**
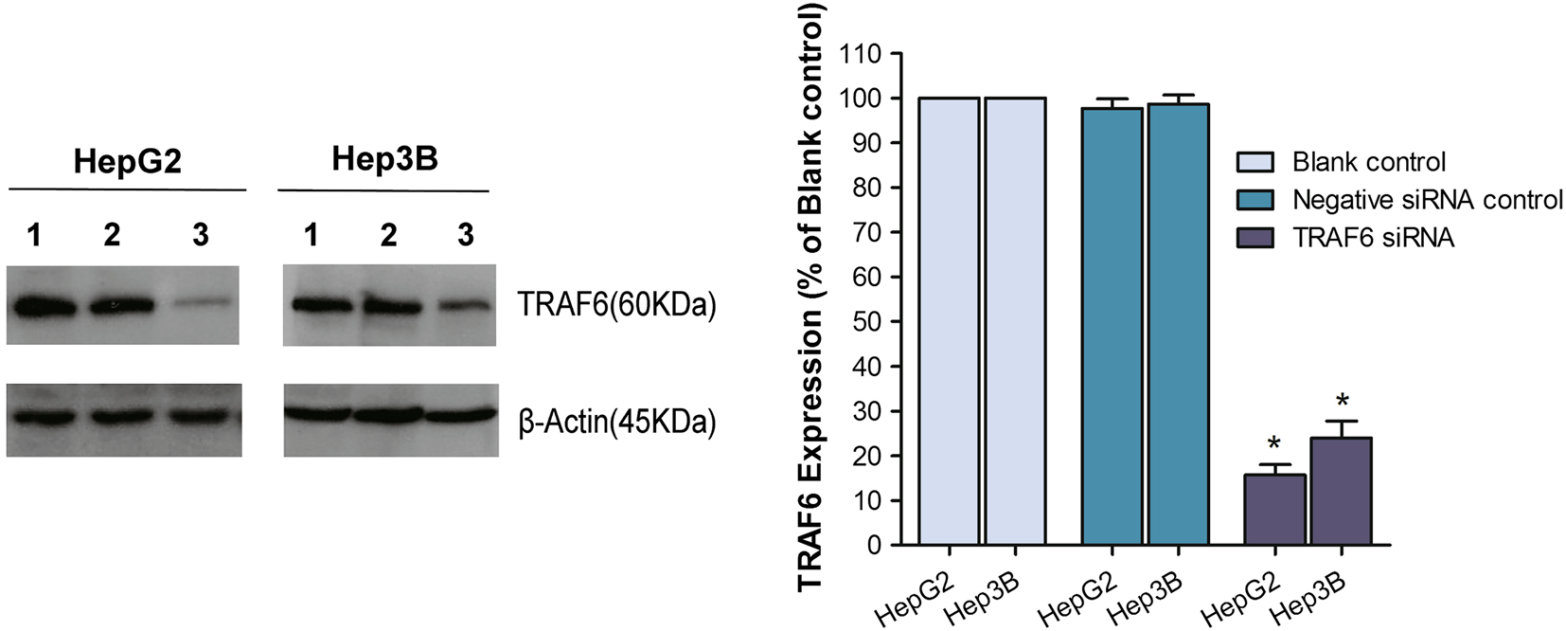
Knock-down effeciency of TRAF6 siRNA in hepatocellular carcinoma HepG2 and Hep3B cells. HepG2 and Hep3B cells were transfected with TRAF6 siRNA and corresponding controls for 10 days. The protein level of TRAF6 was assessed by Western blot (*1*: Blank control, *2*: Negative siRNA control, *3*: TRAF6 siRNA). *P < 0.01

**Fig. 4:**
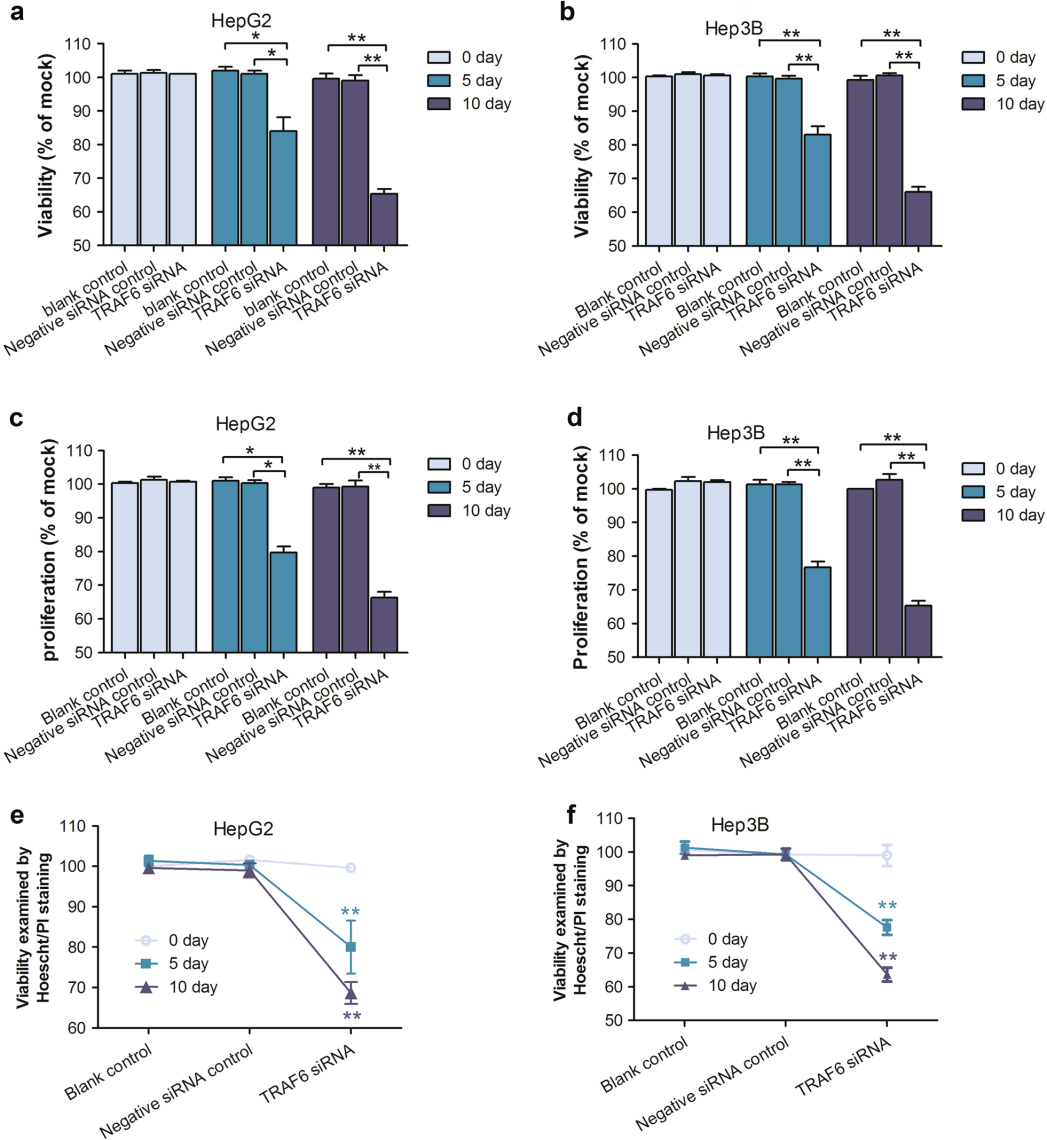
Effect of TRAF6 siRNA on the cell growth of HCC cells. HepG2 and Hep3B cells were transfected with TRAF6 siRNA and corresponding controls for 5 and 10 days. Cell viability was assessed by Cell Titer-Blue kit (**a** HepG2,** b** Hep3B); Proliferation was evaluated with MTS assay (**c** HepG2,** d** Hep3B); Cell viability was also verified by Hoechst/PI double staining (**e** HepG2,** f** Hep3B). *P < 0.01, **P < 0.001

The knock-down efficiency was satisfactory (70–90 %) as assessed by real time RT-qPCR (data not shown) and western blot on the 5th and 10th day post transfection (Fig. 3). To evaluate the effects of TRAF6 on cell viability in the HepG2 and Hep3B cell lines, we conducted Cell Titer-Blue kit and Hoechst/PI analysis. The cell Titer-Blue kit results showed that cell viability was suppressed on the 5th day and 10th day after TRAF6 siRNA transfection on both two cell lines. Moreover, as shown in Fig. 4, cell viability was significantly inhibited on the 5th day and 10th day for both cell lines (HepG2: *P* = 0.004 on the 5th day, *P* < 0.001 on the 10th day; Hep3B: *P* < 0.001 on 5th, 10th day). The results with Hoechst/PI method were in line with results with Cell Titer-Blue kit, however, a bit different. In the HepG2 cells, the statistical difference among blank, negative siRNA control and TRAF6 siRNA groups was only found on the 10th day after transfection (*P* < 0.001) (Fig. 4). In addition, the cell viability after TRAF6 siRNA transfection was obviously reduced on the 5th, 10th day in Hep3B cells (both *P* < 0.001). The effects of TRAF6 on cell proliferation were further analyzed by MTS assay technique, a common technology to confirm cell proliferation status. The MTS assay data revealed that cell proliferation was inactive after being treated with TRAF6 siRNA on the 5th day and 10th day in HepG2 cells as well as Hep3B cells (Fig. 4), with *P* value less than 0.001. Thus, these results suggested that the suppression of TRAF6 reduced the cell growth of HCC HepG2 and Hep3B cells.

### TRAF6 siRNA transfection enhanced the cell apoptosis and caspase activity

**Fig. 5:**
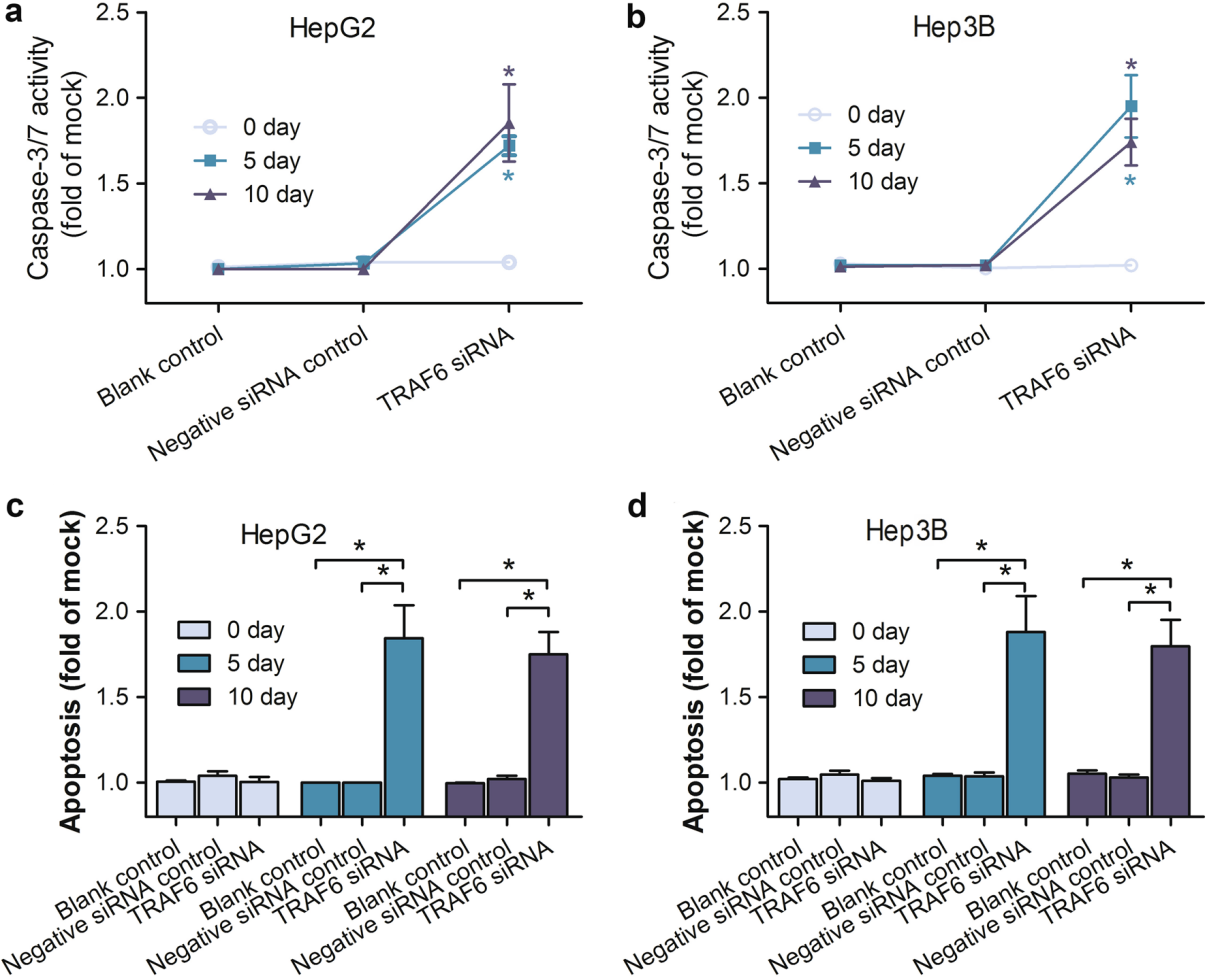
Effect of TRAF6 siRNA on the cell apoptosis of HCC cells. HepG2 and Hep3B cells were transfected with TRAF6 siRNA and corresponding controls for 5 and 10 days. Cell caspase-3/7 activity was assessed by Hoechst/PI double staining (**a** HepG2,**b** Hep3B); Apoptosis was detected by Hoechst/PI double staining (**c** HepG2,** d** Hep3B). *P < 0.01, **P < 0.001

**Fig. 6:**
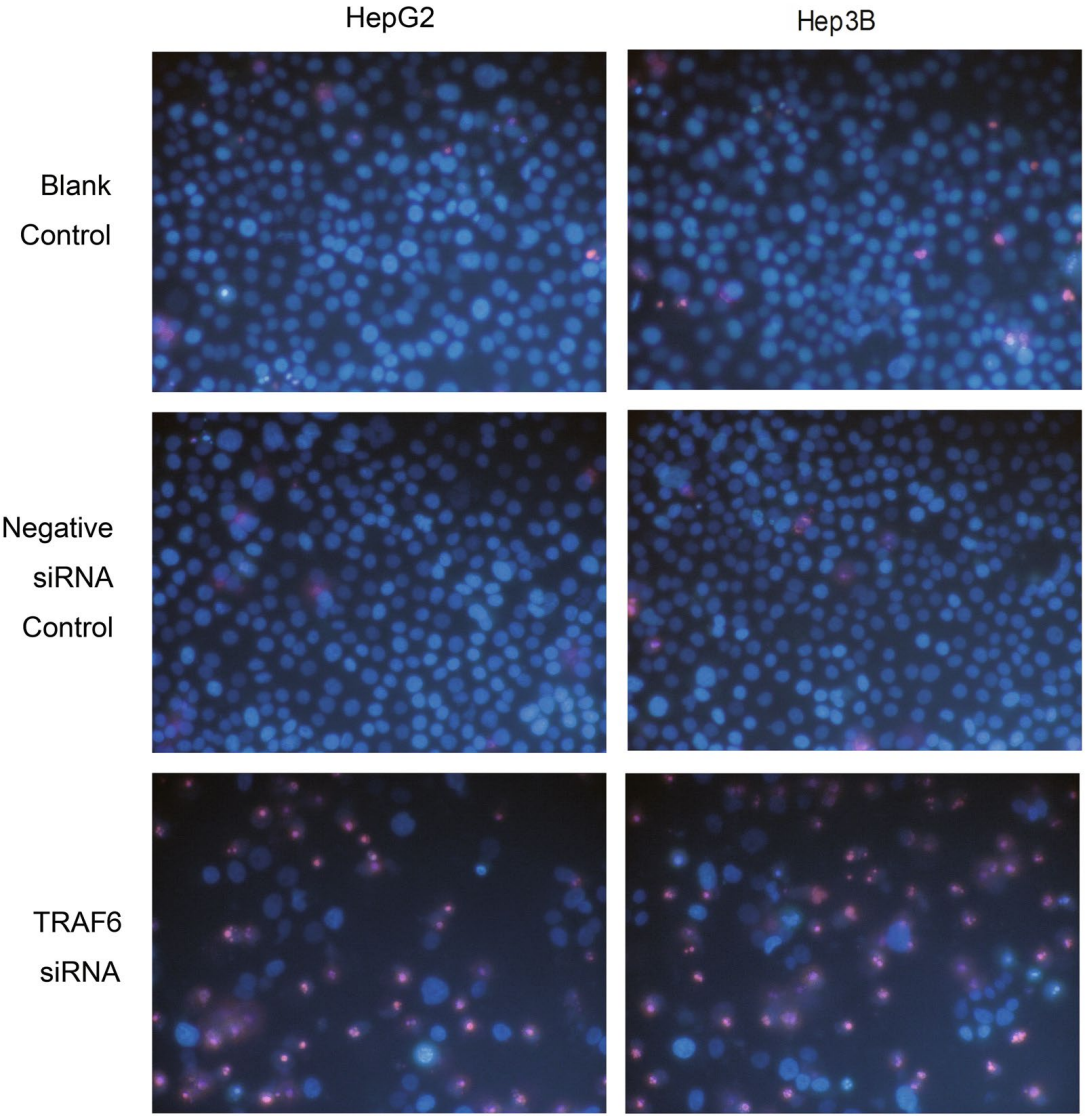
Effect of TRAF6 siRNA on the cell apoptosis of HCC cells assessed by Hoechst/PI double staining. HepG2 and Hep3B cells were transfected with TRAF6 siRNA and corresponding controls for 10 days. Cell apoptosis was detected by Hoechst/PI double staining. ×400

The Hoechst/PI assay was used to investigate the effects of TRAF6 on the cell apoptosis. Cell apoptosis was enhanced after being transfected with TRAF6 on the 5th day (*P* = 0.021) and 10th day (*P* = 0.039) in the HepG2 cell lines, compared with groups of negative siRNA, and blank control (Figs. 5, 6). Similar significant difference of cell apoptosis was seen among groups of TRAF6 siRNA, negative siRNA, and blank control on the 5th day (*P* = 0.019) and 10th day (*P* = 0.045) in the Hep3B cell lines (Figs. 5, 6). Considering the caspase-3/7 activity as reflection of cellular apoptosis, we quantitated the caspase-3/7 activity in both HepG2 and Hep3B cell lines. As shown in the Fig. 5, the activity of caspase-3/7 was significantly higher in HepG2 cells treated with TRAF6 siRNA than groups of blank control and negative siRNA control on the 5th day (*P* = 0.035) and 10th day (*P* = 0.022). Consistent statistical significance was found in the Hep3B cell lines (Fig. 5). The results indicated that TRAF6 could inhibit cell apoptosis and caspase-3/7 activity in HCC HepG2 and Hep3B cells.

## Discussion

HCC is one of the most common, aggressive and lethal cancers in the world [26]. Owing to its burden to human health, early and effective diagnosis is of vital importance. As the genomic researches develop, increasing numbers of novel molecular biomarkers have been regarded as specific diagnosis predictor and targeted therapeutic agents for HCC [27–30]. However, most of those molecular biomarkers for diagnosis and treatment have not been clearly clarified. Additionally, the carcinogenesis of HCC is a multistep process and the mechanism of tumorigenesis and deterioration of HCC has not been fully confirmed. Therefore, we devoted great efforts to look for novel biomarkers of HCC and cast light on the potential role that TRAF6 might play in the genesis and progression of HCC.

Several studies have paid close attention on the role of TRAF6. For instance, Liu et al. proposed that TRAF6 might be considered as a potential target for the treatment of Multiple myeloma (MM) as the receptor activator of nuclear factor-kappaB/receptor activator of nuclear factor-kappa B ligand/tumor necrosis factor receptor-associated factor (RANK/RANKL-TRAF6) signal pathway mediated osteolytic bone lesions [22]. Our previous study got the conclusion that TRAF6 may become a target for diagnosis and gene treatment for lung cancer patients as the TRAF6 had a higher level of expression in lung cancer tissues than in normal lung tissues [31]. Chiu et al. suggested that TRAF6 inhibition might represent a new therapeutic strategy for pancreatic cancer as the down-regulation of TRAF6 led to a remarkable increase in autophagy and apoptosis [32]. However, only Xu Z et al. focused on the relationship between TRAF6 and HCC. They indicated that Cezanne2 interacted with TRAF6 and cleaved the polyubiquitin from TRAF6 substrates whose suppression might have a key role in the HCC malignancy transformation [33]. However, the study was lack of in vivo and in vitro verification.

In the current study, different from previous experiment methods comparing only normal liver tissues and HCC tissues, we examined TRAF6 expression in continuous liver tissues, including normal liver, cirrhosis, para-cancer liver and HCC tissues, which gave us a chance to observe a relatively continuous expressional change of TRAF6 in the genesis of HCC. Positive TRAF6 expression rates were 12.1, 21.6, 36.3, 49.7 % in the normal, cirrhosis, para-cancer and HCC tissues, respectively. The results showed an increasing expression trend in the process of carcinogenesis. Moreover, we performed ROC curve to analyze its diagnostic ability. That we found the AUC of TRAF6 was 0. 595 in HCC, lower than that in lung cancer which was 0.663, indicated the diagnosis value of TRAF6 was moderate for HCC or lung cancer [31]. However, coexistent significant statistical differences were not observed in the dynamic development of liver cancer. Namely, significant differences were only found apart between normal and HCC tissues (*P* < 0.001), cirrhosis and HCC tissues (*P* = 0.002), para-cancer and HCC tissues (*P* = 0.012), normal and para-cancer tissues (*P* = 0.007). This may be due to the limitation of case number. Further larger cirrhosis and normal samples should be enrolled to investigate the differential expression in the dynamic development of HCC.

In order to explain the role of TRAF6 in the HCC angiogenesis, we quantified the expression of VEGF and MVD in this study. No correlation was found between TRAF6 and VEGF, while TRAF6 was associated with MVD closely (r = −0.249, *P* = 0.004). This indicated that TRAF6 might act as a repressor in the angiogenesis of HCC. However, the AUC of MVD was 0.624, lacking high accuracy in predicting the status of micro-vessels. Besides, TRAF6 was connected with metastasis (r = 0.158, *P* = 0.039). Although VEGF and MVD were able to predict vascular metastasis and invasion of HCC [34], the role of TRAF6 in contributing to metastasis did not lie in the aspect of angiogenesis, as indicated above.

To confirm the biological function of TRAF6 in HCC cells, we focused on the application of siRNA interference technology with in vitro experiments, using HepG2 and Hep3B cell lines. We found that cell growth was inhibited in both two cell lines after TRAF6 was knocked-down with siRNA. Since good cell viability is important for cellular material transporting, high expression of TRAF6 might promote cancer growth by keeping cancer cells alive. As is known, cell proliferation and apoptosis play important roles in cancers, we also explored the effects of TRAF6 on cell proliferation and apoptosis by its knock-down, and results showed that the ability of cell proliferation was reduced while the action of cell apoptosis and caspase activity was enhanced after TRAF6 was silenced. Peng et al. reported that knock-down of TRAF6 could suppress cell proliferation and promote cell apoptosis in the glioma cell [35], and Sun and his colleagues found that the TRAF6 level was increased in the colon cancer and its knockdown inhibited cell proliferation but did not improve the survival time [36]. In general, TRAF6 may participate in the cell growth and apoptosis in HCC as well as other cancers being reported.

As for the role that TRAF6 played in cancer invasion and metastasis, a study on esophageal squamous cell carcinoma revealed that TRAF6 promoted migration and metastasis by regulating the RAS pathway [37]. In the analysis of correlations between TRAF6 expression and clinicopathological features, we found that high expression of TRAF6 was associated with metastasis. Although there was no in vitro experiment on the metastasis in this study, based on existing literatures, HCC may be similar with esophageal squamous cell carcinoma where TRAF6 influences cell migration and metastasis, which we will perform experiments to verify.

In summary, our study revealed that the level of TRAF6 was higher in the HCC tissues compared to normal, cirrhosis and para-cancer liver tissues. Meanwhile, TRAF6 expression was related to metastasis and MVD. Most importantly, experiments in vitro showed that TRAF6 could increase the cell viability, promote cell proliferation, and inhibit cell apoptosis and caspase-3/7 activity. All these results elucidated that overexpression of TRAF6 might promote HCC progression. TRAF6 may be a key biomarker in the HCC carcinogenesis and development. It would be beneficial to pay more attention on exploring the regulation mechanisms of TRAF6 in the HCC deterioration in the future.
