# Correction to: Relationship between TRAF6 and deterioration of HCC: an immunohistochemical and in vitro study

**DOI:** 10.1186/s12935-020-1138-x

**Published:** 2020-02-21

**Authors:** Jian-jun Li, Jie Luo, Jing-ning Lu, Xiao-na Liang, Yi-huan Luo, Yong-ru Liu, Jie Yang, Hua Ding, Gui-hui Qin, Li-hua Yang, Yi-wu Dang, Hong Yang, Gang Chen

**Affiliations:** 1 Department of General Surgery, Western Branch, First Affiliated Hospital of Guangxi Medical University, Nanning, 530021 Guangxi Zhuang Autonomous Region People’s Republic of China; 2 Department of Medical Oncology, First Affiliated Hospital of Guangxi Medical University, 6 Shuangyong Road, Nanning, 530021 Guangxi Zhuang Autonomous Region People’s Republic of China; 3 Department of Hepatobiliary Surgery, First Affiliated Hospital of Guangxi Medical University, 6 Shuangyong Road, Nanning, 530021 Guangxi Zhuang Autonomous Region People’s Republic of China; 4 Department of Pathology, First Affiliated Hospital of Guangxi Medical University, 6 Shuangyong Road, Nanning, 530021 Guangxi Zhuang Autonomous Region People’s Republic of China; 5 Department of Pharmacology, School of Pharmacy, Guangxi Medical University, Nanning, 530021 Guangxi Zhuang Autonomous Region People’s Republic of China; 6 Department of Radiotherapy, First Affiliated Hospital of Guangxi Medical University, 6 Shuangyong Road, Nanning, 530021 Guangxi Zhuang Autonomous Region People’s Republic of China; 7 Department of Ultrasonography, First Affiliated Hospital of Guangxi Medical University, 6 Shuangyong Road, Nanning, 530021 Guangxi Zhuang Autonomous Region People’s Republic of China

## Correction to: Cancer Cell Int (2016) 16:76 10.1186/s12935-016-0352-z

Following the publication of the original article [1], the authors reported that they had supplied the incorrect figure 6 for publication. The correct Fig. 6 is given in this correction article. The results and conclusions described therein are not affected by these corrections. The authors sincerely apologize for the error. This has now been included in this correction article.

**Fig. 6:**
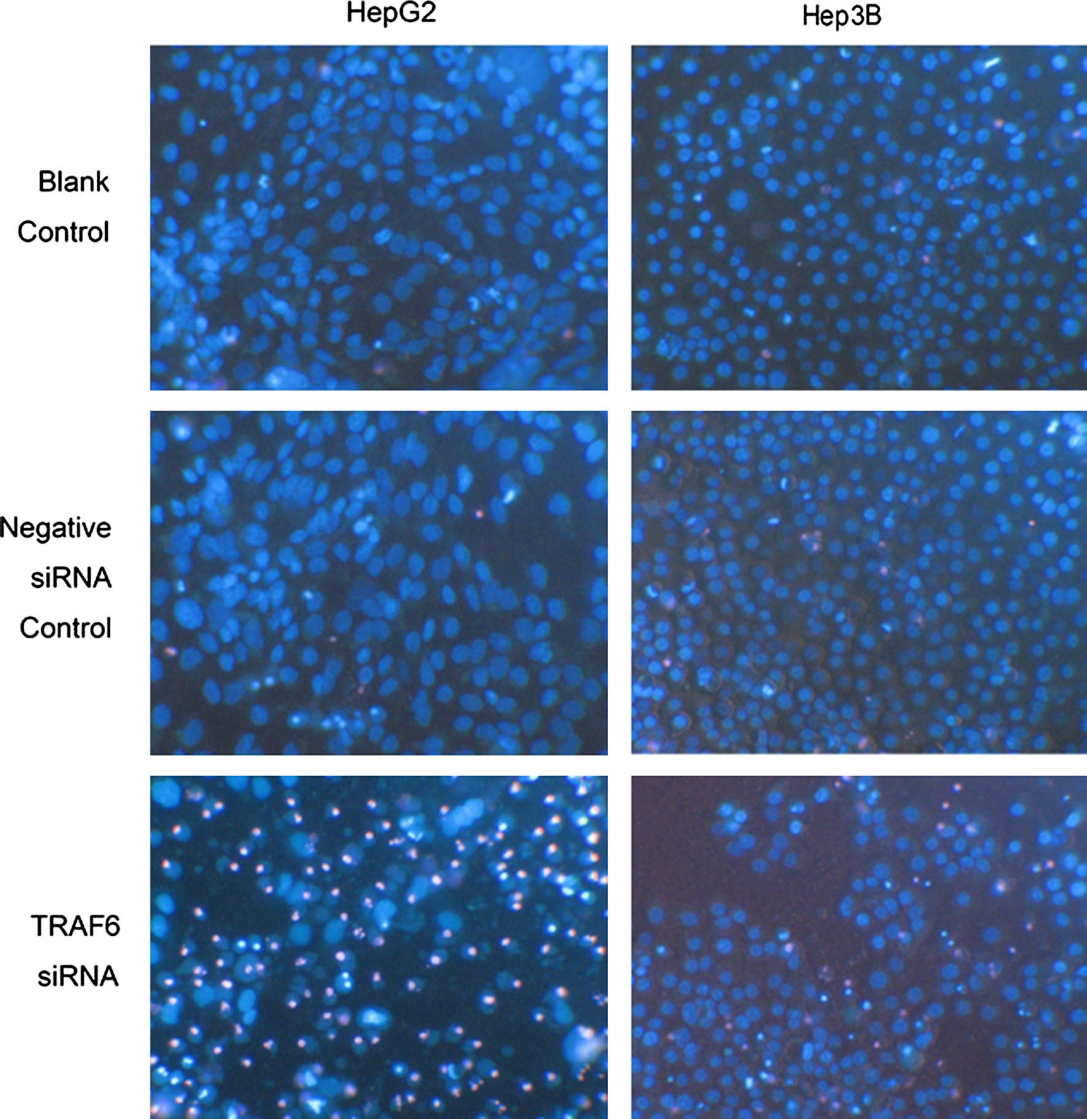
Effect of TRAF6 siRNA on the cell apoptosis of HCC cells assessed by Hoechst/PI double staining. HepG2 and Hep3B cells were transfected with TRAF6 siRNA and corresponding controls for 10 days. Cell apoptosis was detected by Hoechst/PI double staining ×400
